# Coronavirus Disease 2019: Clinics, Treatment, and Prevention

**DOI:** 10.3389/fmicb.2021.761887

**Published:** 2021-11-11

**Authors:** Francesco Robert Burkert, Lukas Lanser, Rosa Bellmann-Weiler, Günter Weiss

**Affiliations:** Department of Internal Medicine II, Infectious Diseases, Immunology, Rheumatology, Pneumology, Innsbruck Medical University, Innsbruck, Austria

**Keywords:** COVID-19, pandemic, SARS-CoV-2, epidemiology, hyperinflammation, PCR testing, antigen tests, transmission

## Abstract

The coronavirus disease 2019 (COVID-19) pandemic, caused by a novel severe acute respiratory syndrome coronavirus-2 (SARS-CoV-2), emerged at the end of 2019 in China and affected the entire world population, either by infection and its health consequences, or by restrictions in daily life as a consequence of hygiene measures and containment strategies. As of September 2021, more than 231,000.000 infections and 4,740.000 deaths due to COVID-19 have been reported. The infections present with varied clinical symptoms and severity, ranging from asymptomatic course to fatal outcome. Several risk factors for a severe course of the disease have been identified, the most important being age, gender, comorbidities, lifestyle, and genetics. While most patients recover within several weeks, some report persistent symptoms restricting their daily lives and activities, termed as post-COVID. Over the past 18months, we have acquired significant knowledge as reflected by an almost uncountable number of publications on the nature of the underlying virus and its evolution, host responses to infection, modes of transmission, and different clinical presentations of the disease. Along this line, new diagnostic tests and algorithms have been developed paralleled by the search for and clinical evaluation of specific treatments for the different stages of the disease. In addition, preventive non-pharmacological measures have been implemented to control the spread of infection in the community. While an effective antiviral therapy is not yet available, numerous vaccines including novel vaccine technologies have been developed, which show high protection from infection and specifically from a severe course or death from COVID-19. In this review, we tried to provide an up-to-date schematic of COVID-19, including aspects of epidemiology, virology, clinical presentation, diagnostics, therapy, and prevention.

## Introduction

At the end of 2019, many people in Wuhan, China, developed signs of a hitherto unknown infection (Huang et al., 2020). Hospitalized individuals presented primarily with fever, cough, and muscle pains, many of them showed abnormalities in the chest computed tomography (CT) and 29% developed acute respiratory distress syndrome (ARDS; Huang et al., 2020). A novel coronavirus was identified as the causal agent (Wenjie et al., 2020) and dubbed as severe acute respiratory syndrome coronavirus 2 (SARS-CoV-2; Gorbalenya et al., 2020). The disease itself was called coronavirus disease 2019 (COVID-19). The infection then spread throughout the entire world and was declared a pandemic by the WHO on March 11, 2020. Due to the contagiousness of the virus, many nations attempted to flatten the epidemiological curve and avoid overburdening of the healthcare systems by enforcing specific hygiene measures, social distancing, mask wearing, travel restrictions, closing of borders, national lockdowns, and mandatory quarantine for the infected and their contact persons (Baker et al., 2020; Thu et al., 2020). During the course of the pandemic, extensive studies analyzed the virus, the epidemiology, immunology, and pathophysiology of COVID-19 and tried to establish optimized diagnostic and prevention strategies. In addition, major efforts were undertaken to identify targeted therapies by re-purposing existing drugs or by applying existing knowledge from best clinical practice of other respiratory infectious diseases to the treatment of COVID-19. Finally, by using different and even novel strategies, numerous effective vaccines have been developed within a surprisingly short period of time. In this review, we aim to provide an overview on the complex topic that is COVID-19, focusing on epidemiology, virological, and immunological knowledge as well as on clinical aspects and possibilities for prevention and therapy.

## Epidemiology

### Origin/Reservoir

The viruses classified as coronaviridae, first discovered at the beginning of the twentieth century, are single-strand RNA viruses that have their primary animal reservoir in bats and birds (Woo et al., 2012). The reservoir animal, while carrying the virus, seldom develops a life-threatening, symptomatic infection (Banerjee et al., 2020). The virus may, however, replicate inside the host when its immune response is diminished, leading to a large number of random mutations which may generate variants capable of infecting other species including humans. This event is referred to as a cross-species spillover event. The spillover event for SARS-CoV-2 is believed to have happened sometime in 2019, although the definitive animal origin, whether snake or bats, remains elusive thus far.

### World Dissemination

The index patient for COVID-19 was admitted to a Chinese hospital in the province of Hubei at the beginning of December 2019 (Huang et al., 2020). Shortly after his admission, a cluster of 40 patients, of which 27 with close contact to the Huanan animal market, also required hospitalization due to COVID-19 associated dyspnea (Huang et al., 2020). The index patient lived alone; no certain epidemiological connection could be established to the Wuhan market outbreak. As local and then international health authorities endeavored to identify the pathogen responsible for this hitherto unknown infection, the virus that would become known as SARS-CoV-2 began to stealthily spread. Confirmed cases appeared first in Asia (World Health Organization, 2020) and then in the American continent (Center for Disease Control and Prevention, 2020). At the end of January, the virus reached Europe (Stoecklin et al., 2020) and Australia (Price et al., 2020). The first confirmed case in Africa was reported on the February 14, 2020 (Medhat and El Kassas, 2020). As COVID-19 continued spreading throughout the globe, the WHO declared a pandemic on March 11, 2020. Most nations managed to flatten the epidemiological curve during the first wave of infections by implementing different non-pharmacological interventions including physical distancing, mask wearing, specific hygiene measures, contact tracing, quarantine, travel restriction, and even nationwide lockdowns (Saez et al., 2020; Valentowitsch, 2020). Of interest, infection numbers declined during summer and increased during the cold season in the Northern Hemisphere which is well known also for other respiratory viruses (Dzien et al., 2020); however, virus circulation was continuously present from the onset of the pandemic. Of note, as this was the greatest pandemic since the Spanish flu more than 100years ago, no coordinated strategies or specific recommendations for the control of the pandemic and its world-wide spread have been developed or implemented either by different states, communities such as the European union or international health organization such as the WHO. As of September 2021, more than 231 million cases of infection have been documented (Center for Systems Science and Engineering at Johns Hopkins University, 2021), and more than 4.7 million SARS-CoV-2-associated fatalities were reported by the European Center of Disease Prevention and Control.

## Virology

### Introduction

The SARS-CoV-2, reportedly first isolated at the beginning of 2020 in China (Wenjie et al., 2020), is one of the coronaviridae, a family of positive-sense single-strand RNA viruses first associated with avian respiratory infections in the 1930s (Schalk, 1931).

Coronaviridae are made up of four distinct genera (alpha, beta, gamma, and delta), with SARS-CoV-2 belonging to the genus of beta-coronaviridae.

Severe acute respiratory syndrome coronavirus-2 is believed to have evolved into its present state in bats, the original animal reservoir, and may have passed through an intermediate host before being finally transmitted to humans (Yuan et al., 2020).

Severe acute respiratory syndrome coronavirus-2 is not the first beta-coronavirus to cause pulmonary disease; coronaviruses have been associated with respiratory infections since the sixties of the last century, when the human coronavirus OC43 was identified as one of the pathogens responsible for common cold. Other noteworthy predecessors include the severe acute respiratory syndrome coronavirus 1 (SARS-CoV-1) and the Middle East respiratory syndrome coronavirus (MERS-CoV), responsible, respectively, for the SARS epidemic and the MERS outbreak (De Wit et al., 2016).

Severe acute respiratory syndrome coronavirus-2 can attack human cells through its viral spikes, capable of interfacing with the human angiotensin converting enzyme 2 (ACE2) receptor (Davidson et al., 2020). Many different human tissues, including the alveolar linings in the lung, vascular endothelia, and intestinal mucous membranes, express this receptor (Hamming et al., 2004). After attaching to the ACE2 receptor, the virus penetrates into the cell and uses its resources to multiply.

Our article is written primarily from the clinical perspective and provides only a superficial overview on the topic of pathophysiology, for deeper insight into the rapidly evolving field of virology, immune response, and immune escape of COVID-19, we refer to recently published and emerging reviews specifically dealing with this fascinating topic (Harrison et al., 2020; Yuki et al., 2020). Our review intends to summarize COVID-19 from a practical and clinical perspective; the included information on virology and clinical pathology is necessary to better understand differences in clinic presentation, complications, and different treatment principles.

### Transmission and Viral Mutants

The transmission of the SARS-CoV-2 virus occurs mainly from person-to-person through exhaled droplets, specifically with closer contacts (<1m distance) and with exposure time of several minutes. Infected individuals develop symptoms after a mean incubation period of 5–6days, and the 95% for development of symptomatic infection after exposure was estimated between 10 and 14days (Linton et al., 2020; McAloon et al., 2020; Tindale et al., 2020).

As in most viral infections, patients are contagious upon onset of the symptomatic phase of the disease, when they can excrete larger quantities of virus by coughing, sneezing and overall dispersing of large quantities of bodily fluids. However, pre-symptomatic patients are contagious 2–3days before they begin feeling sick, thereby greatly enhancing the number of potentially infected contacts and making outbreak control more difficult (Gandhi et al., 2020; Kucharski et al., 2020; Tindale et al., 2020).

After SARS-CoV-2 has been incorporated, mostly *via* inhalation of infected droplets, it can invade host cells and induce its own replication. However, not all exposures to viruses lead to symptomatic infection. The number of viruses inhaled, on the one hand, and the activity of the host’s innate immune system, on the other, determine whether the infection will become manifest or not (Casadevall and Pirofski, 2018). Therefore, not all subjects having been in contact with the virus or even tested positive for SARS-CoV-2 by PCR are able to transmit the infection, as they may have cleared the virus through local immune responses which are mainly mediated by pathogen recognition receptor inducible immune activation pivotally involving formation of type I interferons (Vabret et al., 2020). The reasons for this are thus either low viral number incapable of establishing infection and/or a potent immune response which can neutralize higher number of viruses. Likewise, such individuals may not develop immune memory to this infection (Gaebler et al., 2021). On the other hand, high viral numbers or mutants with an increased infectivity (such as the B.1.1.7 or the B.1.617.2 subtypes) and/or a less effective local immune response result in establishment of infection. It is currently assumed that between 67 and 90% of positively tested people or of those who tested positive for anti-SARS-CoV-2 antibodies later on have experienced symptoms, while the other individuals remained asymptomatic (He et al., 2021).

Among infected people developing symptoms, an estimated 80–85% experience mild flu-like symptoms, such as fever, cough, myalgia, fatigue, or hyposmia, and most of them recover within days or a few weeks (Berlin et al., 2020). The remaining 15–20% suffer either from more severe symptoms and/or a prolonged course of the disease with the development of dyspnea or organ damage which most frequently develops 7–10days after onset of symptoms. About half of these patients may require hospitalization, and approximately 20% of those (2–5% of all infected patients) may need treatment at an intensive care unit (ICU; Berlin et al., 2020). Mortality rates greatly vary depending on risk factors, age, evaluation of true prevalence of infection by test strategies and access to medical treatment, but it is estimated to lie between 0,3 and 3% of all infected individuals (Gautret et al., 2020).

The estimated basic reproduction number for SARS-CoV-2 (R0) at the time of the original Wuhan outbreak was 2.6–3.8 (Sanche et al., 2020), meaning that one infected individual could go on to infect 2.6–3.8 further contacts (European Centre for Disease Prevention and Control, 2021a). By comparison, R0 of up to 18 and 2.1 has been calculated for measles and influenza, respectively (Coburn et al., 2009; Guerra et al., 2017).

At the time of writing, mutations of the SARS-CoV-2 virus have been identified which may either be more contagious, partly escaping the immune control of previously infected or vaccinated people or causing more severe disease (Kuzmina et al., 2021; Lauring and Hodcroft, 2021). One of the first mutated viruses described, the D614G variant, caused by a single nucleotide substitution in the genetic material coding for the viral protein spike, was discovered in February 2020. This mutation increased viral fitness and D614G rapidly overcame the wildtype as the most frequent viral variant (Korber et al., 2020). Another mutation resulted in a new variant of concern (VOC), which was first detected in Great Britain in September 2020 and then dubbed VOC-B.1.1.7 or alpha variant. This variant was reported to be more transmittable by 35–50% as compared to the wildtype virus due to higher affinity of the virus caused by a mutation in the receptor binding site of the viral spike protein but also by its ability to block the anti-viral immune response (Jones et al., 2021; Thorne et al., 2021). However, whether this variant also causes more severe disease or case fatalities remains controversial (Frampton et al., 2021; Graham et al., 2021; Grint et al., 2021).

Subsequently, numerous other variants of concern evolved in different regions of the world and spread according to either higher transmissibility and/or immune escape, meaning that those variants, like the ones first identified in Great Britain (B 1.117+ E484K, alpha), South Africa (B 1.351, beta) Brazil (P1, gamma), United States (B 1.427, kappa) or India (1.617, delta), are less susceptible to neutralization by monoclonal antibodies or antibodies generated during primary infection or antibodies produced following vaccination (Corti et al., 2021; European Centre for Disease Prevention and Control, 2021b).

Primary routes for infection are respiratory droplets larger than 5μm, which can be propelled up to 6m by coughing or sneezing or crying/singing (Fennelly, 2020; Prather et al., 2020). These droplets are easily affected by gravity due to their size, and therefore normally precipitate shortly after expulsion. The viruses contained in these larger droplets target bronchial epithelial cells primarily in the upper airways (Fennelly, 2020). Due to the large amount of infectious material contained in the expelled bodily fluids, direct exposure to an infected person for more than 15min at a distance of less than 1m, especially in a closed, unventilated environment, is a major risk factor for acquisition of infection (Chu et al., 2020; Kucharski et al., 2020). However, individuals infected with more contagious variants such as the alpha or delta VOCs may prove infectious even for encounters shorter than 15min or at distances greater than m (Jones et al., 2021). Importantly, the transmission risk is directly linked to the intensity of contacts, ranging from close to 50% among spouses to 7–18% secondary attack rates in household members, to almost 0% among distant contacts (Böhmer et al., 2020; Jing et al., 2020; Madewell et al., 2020). Of note, secondary infection rates from asymptomatic individuals are described being around 1% (Madewell et al., 2020; Qiu et al., 2021). Importantly, most of those data have been generated by epidemiological studies of the wildtype viral strain, and secondary attack rate may likewise change according to the higher infectivity of VOCs.

Infection through airborne droplets smaller than 5μm, capable of lingering in the air for longer amounts of time, is rare but also possible (Fennelly, 2020). These droplets, produced by talking, intensive ventilation, singing, or wind instruments, are easily dispersed by air currents and can be quickly inactivated by direct sunlight (Schuit et al., 2020). For this reason, assemblies of many people in poorly ventilated environments, such as in cruise ships, churches, concert halls, offices, or retirement homes, may cause secondary infections and outbreaks (Leclerc et al., 2020). SARS-CoV-2 is also suggested to be transmitted by airborne droplets through the ocular surface, especially in hospitals and medical staff (Chen et al., 2021b).

Transmission through fomites such as door handles, utensils, and public transportation is possible but not as well documented as airborne or droplet transmission. Nonetheless, correct hand hygiene and surface disinfection, as documented in other major viral epidemics, can assist in reducing the number of new infections (Liu et al., 2016).

Due to yet unknown factors, some individuals, known as super-spreaders, may infect more people than the usually calculated R0 value. However, while some believe super-spreaders to emit more infectious particles than average, it is more likely that the dissemination of the SARS-CoV-2 epidemic has been accelerated by key super-spreading events, such as religious gatherings or cruises or in nightclubs and bars (Majra et al., 2021; Muller et al., 2021).

Some studies have detected SARS-CoV-2 in the stool of infected patients, as well as in sewage samples from various hospitals and cities affected by the epidemic (La Rosa et al., 2020). This implies the theoretical possibility of fecal-oral transmission or transmission by aerosolized particles (Carvalho et al., 2020; Effenberger et al., 2020). The presence of the virus in sewage may be used for monitoring and early detections of further waves of infection (Ahmed et al., 2021).

While the sexual route is not a commonly described infection route for SARS-CoV-2, the virus has been detected in semen and, due to the intimate nature of sexual contact, the transmission of the infection through intercourse appears possible (Patrì et al., 2020).

## Clinical Presentation and Course of the Infection

### Incubation Period

There has been considerable controversy regarding the incubation time for infection with SARS-CoV-2. The data acquired from the first documented outbreak in China (Guan et al., 2020) reported development of symptoms after a median of 4days following exposure. While an estimated 97.5% of infected patients are expected to develop symptoms within 11.5days (Lauer et al., 2020), incubation times of up to 13days have been observed in the elderly (Kong, 2020).

A systematic review and meta-analysis of the published observational research estimated a mean incubation time of 5.8days and a median incubation time of 5.1days, with a 95th percentile of 11.7days (McAloon et al., 2020).

Due to the increased economic and social burden of extensive quarantine and self-isolation practices, shortened quarantine models have been proposed for people post-exposure. Herein, individuals could terminate isolation after 7days, if remaining completely asymptomatic and being tested negative for COVID-19 by real-time PCR (RT-PCR) or antigen test after the average incubation time (Fox et al., 2021).

Based on the available data, a 14-day quarantine for citizens exposed to COVID-19 should adequately reduce the possibility of further viral spreading (Wölfel et al., 2020). A 10-day quarantine, while sufficient for most of the general population, may be sufficient in the majority of cases, but may prove too short for selected elderly or immunocompromised patients who may still develop symptoms up to 3–4days after the end of contact isolation due to delayed viral clearance. Shortened protocols can be applied if the necessary infrastructure for testing is present. Duration of quarantine should therefore be decided on an individual basis.

### Symptoms and Signs at Presentation

The patients from the Huanan fish market outbreak initially showed typical symptoms of a flu-like infection. These included fever, cough, myalgia, and fatigue and, less frequently, headache, hemoptysis, and diarrhea (Huang et al., 2020). As the pandemic spread and more data were made available, it became clear that the clinical picture was more complex than hitherto imagined.

The reported percentage of asymptomatic infections with COVID-19 varies widely. In February 2020, the Chinese Center for Disease Control (CCDC) shared a large case series, reporting on 72,314 COVID-19 patients infected in the first wave of the pandemic (Wu and McGoogan, 2020). Of those, 1% were tested positive by PCR but experienced no symptoms. In the outbreak on the Diamond Princess cruise ship, 17.9% of the infected passengers reported to have remained asymptomatic (Mizumoto et al., 2020). In a seroprevalence study in Ischgl, Austria, 11.6% of 197 seropositive residents reported no symptoms (Lehmann et al., 2021).

In symptomatic patients, the disease can be mild, severe, or critical (Wu and McGoogan, 2020).

Patients experiencing a mild disease course most frequently report fever, cough, and fatigue, but may also experience myalgia, headaches, increased sputum production, and sore throat (Fu et al., 2020); many infected individuals report nausea, vomiting, or diarrhea, symptoms potentially explained by the abundance of ACE2 in the gastrointestinal tract (Hamming et al., 2004). In addition, a varying percentage of subjects report a loss of smell or taste which tends to persist longer than 3months (Stokes et al., 2020; Rass et al., 2021).

While many patients recover in a couple of weeks, 10–20% may develop a severe disease with progressive pneumonia and dyspnea or other complications around the 7–10thday of symptomatic infection often requiring hospitalization (Berlin et al., 2020; Sonnweber et al., 2020; Stokes et al., 2020). Among those, 2–5% develop critical disease requiring ICU admission due to sustained hypoxia, respiratory or circulatory failure, shock or multiple organ dysfunction syndrome (MODS; Oberfeld et al., 2020; Stokes et al., 2020; Wu and McGoogan, 2020).

### Typical Course of the Disease

**Figure 1:**
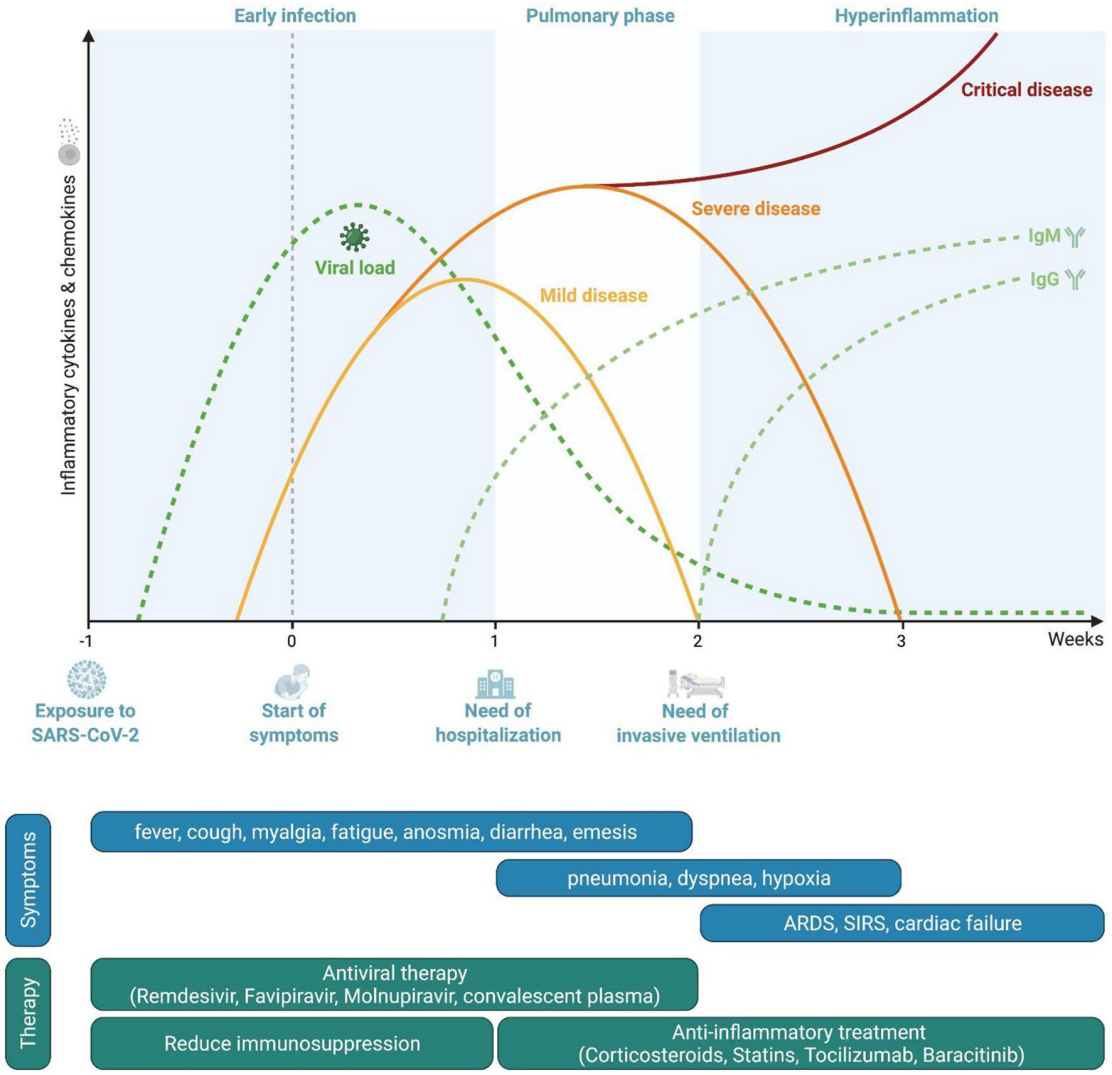
Pathophysiological course of the disease and phase-oriented treatment principles. Representation of viral loads and serological response during coronavirus disease 2019 (COVID-19). Timeframe for disease severity as well as phase-related symptoms and therapeutic options.

The current paradigm divides the course of infection into three phases (Romagnoli et al., 2020; Figure 1).

After the infection of the host has occurred, SARS-CoV-2 accesses vulnerable cells through the ACE2 receptor, resulting in viral amplification and subsequently in viremia targeting multiple organs. In this first “early infection” phase, patients experience mild symptoms such as fever, myalgia, headache, or fatigue but also hyp-/anosmia or diarrhea, depending on viral load, the local immune responses and individual distribution of ACE2. In this early phase, innate immune responses include mainly the production of type I interferons (Vabret et al., 2020). Impaired type I interferon signaling and autoantibodies directed against interferons are associated with a severe course of the infection (Bastard et al., 2020; Zhang et al., 2020a).

If local clearance of infection by type I interferons and phagocytosis and clearance of infected cells by macrophages are not successful either due to high viral loads and/or diminished immune response, subsequent activation cascades of innate and adaptive immune cells occur which result in systemic inflammation. Specifically, an efficient antigen presentation and subsequent activation of T-lymphocytes is a pre-requisite for infection control and resolution of inflammation (Olwenyi et al., 2020). This secondary phase may be associated with involvement of organs like the lung, leading to an accumulation of inflammatory cells, vasodilation with extravasation of fluids and development of infiltrates resulting in pneumonia. Depending on the severity of pulmonary inflammation and impairment of alveolar gas exchange, hypoxia may occur with patients reporting shortness of breath. Systemic inflammation also has a pro-thrombotic effect due to activation of the coagulation cascade but also due to expression of anchor proteins on the surface of endothelial cells which promote attachment of platelets and development of thrombotic clots (Gupta et al., 2020). About 7–10days after the onset of the disease, individual factors in the host’s immune system determine whether the disease may advance to the third phase.

If control of infection is not achieved or the immune response is improper, a “hyperinflammation phase” develops which shares many parallels to macrophage activation syndromes (Webb et al., 2020), characterized by, among others, overwhelming activation of T-lymphocytes with increased formation of interferon-gamma, neutrophils, and dysfunctional hyperactivated monocytes (Olwenyi et al., 2020; Schulte-Schrepping et al., 2020; Vabret et al., 2020; Bellmann-Weiler et al., 2021). Excessive immune activation and cytokine/radical formation result in tissue damage and eventual organ failure, such as ARDS in the case of the lung (Romagnoli et al., 2020). The extensive endothelial activation may lead to life-threatening complications such as disseminated intravascular coagulopathy, pulmonary embolism, acute coronary syndrome, or stroke (Bikdeli et al., 2020). Extensive systemic inflammation may compromise multiple organ systems, leading to MODS (Robba et al., 2020).

### Risk Factors for Severe Disease

Since the beginning of the pandemic, major emphasis has been placed on the search for risk factors for both, severe disease, and death, as well as toward the identification of biomarkers which predict an adverse course of the infection.

Two very important predictors for severe disease are age and gender. While only 1.04% of infected patients aged 20–29 required hospitalization, the percentage progressively increased in every age group and reached the maximum of 18.4% in patients older than 80 (Verity et al., 2020). In addition, advanced age is associated with higher risk of pulmonary failure, need for mechanical ventilation and death (Karagiannidis et al., 2020; Wu et al., 2020). Regarding gender, while the sexes do not differ in susceptibility to infection, a significantly higher percentage of men experience severe or critical COVID-19 disease than women (Jin et al., 2020; Kragholm et al., 2020). This may be due to genetic differences as the X-chromosome harbors several innate immunity genes, hormonal differences specifically testosterone levels, but also to higher prevalence of comorbidities or perhaps differences in lifestyle, with men more frequently engaging in smoking or alcohol consumption (Mauvais-Jarvis et al., 2020; Lanser et al., 2021c). Nonetheless, studies correcting for these factors still determined an independent association of male sex with more severe disease (Kragholm et al., 2020).

Presence of comorbidities also correlate with severe disease courses. Obesity, while frequently being associated with ICU admission and necessity for mechanical ventilation, does not correlate with elevated mortality. Other frequent comorbidities, especially hypertension, diabetes, respiratory diseases, cerebrovascular disease, cancer, and chronic kidney disease, significantly influence the probability of severe disease with ICU admission, mechanical ventilation, and potentially death (Zhou et al., 2020). Patients with chronic liver disease also show higher rates of hospital admission and mortality (Wang et al., 2021). A meta-analysis also associates immunosuppression with severe disease course, even though the correlation did not reach statistical significance (Gao et al., 2020).

Even genetics seem to play an important role in the development of severe disease and mortality. The immune-mediated inflammation in COVID-19, while important in locally combating pathogens, may also damage innocent bystander host tissue, potentially exacerbating organ damage. Interferons contribute by modulating the immune response to preserve native tissue. Zhang et al. (2020a) detected mutations in genes involved in the regulation of IFN type III and I in patients with severe COVID-19 disease. A genome wide association study identified six genetic risk factors for severe disease, and five of those are linked to T-lymphocyte function and its interaction with antigen presenting cells (Ellinghaus et al., 2020). In addition, blood group A appears to increase the risk for severe infection (Ellinghaus et al., 2020).

Several biomarkers have been identified which are associated with either a poor prognosis or a higher probability of advancing from infection to severe or critical disease. It has been shown that among other risk factors lymphopenia, specifically reduced numbers of CD4+ and CD8+ T-lymphocytes, high numbers of neutrophils, dysfunctional monocytes, higher circulating concentrations of immune biomarkers such as interleukin 6 (IL-6), IFN-gamma or neopterin as well as higher ferritin levels or presence of anemia at diagnosis were associated with an adverse outcome (Bellmann-Weiler et al., 2020, 2021; Manson et al., 2020; Schulte-Schrepping et al., 2020; Zhang et al., 2020b; Lanser et al., 2021b).

### Organ Involvement in Complicated Infection

**Figure 2:**
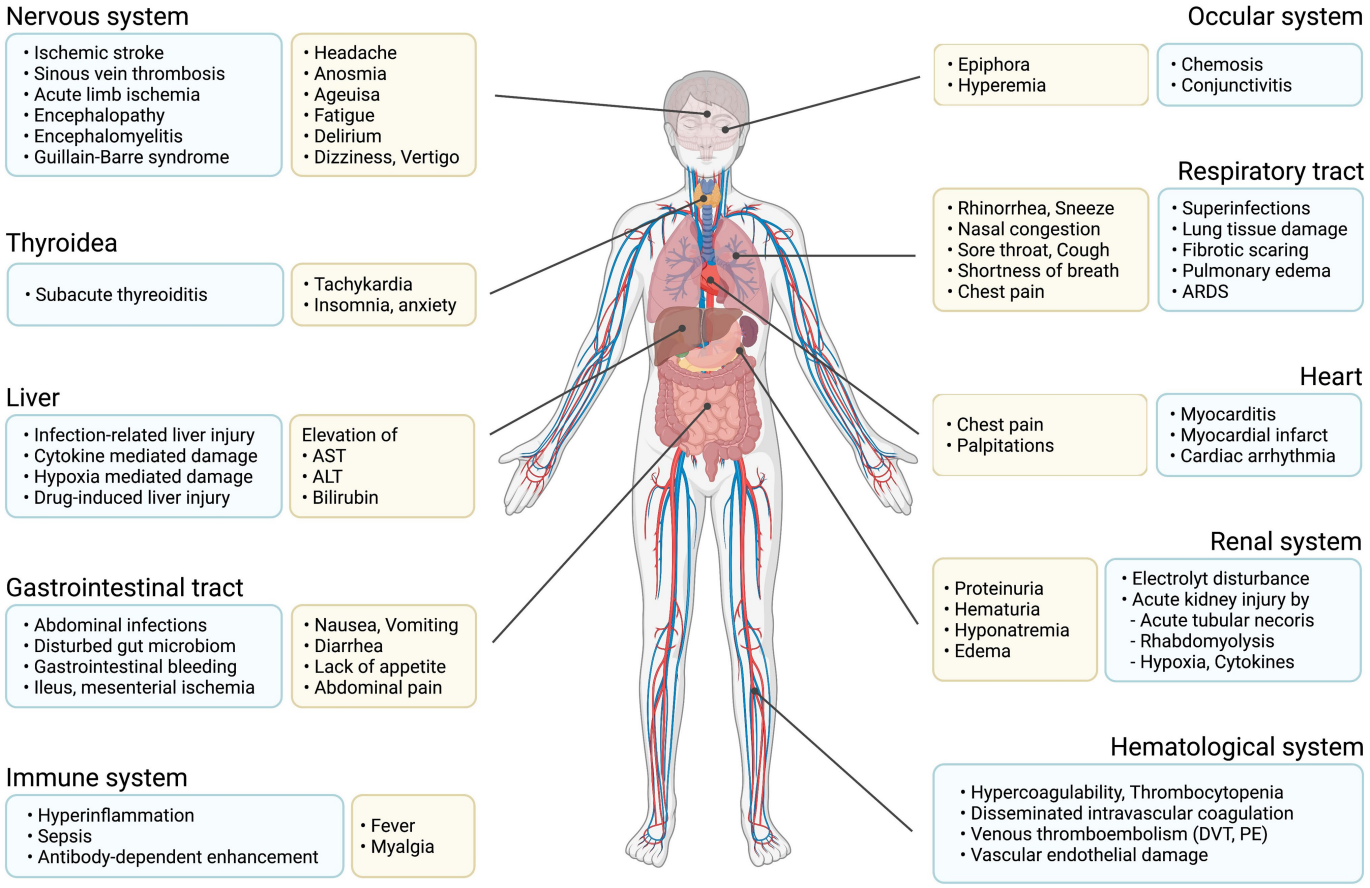
Main organ complications due to COVID-19. Graphical representation of possible symptoms and organ damage caused by COVID-19.

Flu-like symptoms and viral pneumonia are not the only possible consequences of an infection with SARS-CoV-2. Due to the expression of ACE2 receptors in multiple tissues, complications may arise in both mild and severe infections and affect different organs (Hamming et al., 2004; Figure 2).

The most frequent complication, caused by virus- and inflammation-mediated tissue damage, is progressive lung disease with drastic reduction of lung functional capacity, as a consequence of viral pneumonia and infection driven inflammatory infiltrates in the alveoli (Olwenyi et al., 2020). With progressive reduction of the gas-exchange surface, as well as reduced lung compliance, patients may develop hypoxia, shortness of breath, bilateral pulmonary infiltrates and respiratory deterioration culminating in respiratory failure with ARDS (Wang et al., 2020a). Based on clinical experience not all patients with hypoxia have an increased respiratory rate, thus pulse oximetry is necessary to reliably identify subjects with hypoxia. Hypoxia often warrants non-pharmacological interventions with oxygen or high flow oxygen therapy, non-invasive mechanical ventilation (NIV), intubation with invasive mechanical ventilation, and extra corporal membrane oxygenation (ECMO) in some cases (Karagiannidis et al., 2020).

Specifically, patients requiring longer hospitalization and invasive procedures but also those receiving immuno-suppressive therapy are at an increased risk for secondary infections. These may occur in 7.2% of cases and are primarily caused by bacteria; however, secondary infections with mold fungi including Aspergillus and Mucor species have also been described (Garcia-Vidal et al., 2021). The most frequent secondary infections are nosocomial or ventilator associated pneumonia and bacteremia, respectively, observed in 25% and 14.9–36.3% of cases, followed by urinary tract infections (in 8.5–27.3% of cases), and sporadic, followed by urinary tract infections and abdominal infections (Chong et al., 2021; De Santis et al., 2021; Garcia-Vidal et al., 2021).

An increase in venous thromboembolic occurrences, such as deep vein thrombosis (DVT) in 20% of cases or pulmonary embolism (PE) in 13% of cases, has been observed in COVID-19 infections (Malas et al., 2020). While most of these complications emerge in severe disease, sporadic events may also be facilitated in mild infections (Klok et al., 2020; Levolger et al., 2020). This may be partly due to the expression of ACE2 receptors on vascular endothelia, as well as the pro-thrombotic state and expression of adhesion molecules on endothelia induced by systemic inflammation (Hamming et al., 2004; Bikdeli et al., 2020).

Arterial thromboembolic events, while less frequent than DVT or PE, are found in a smaller percentage of COVID-19 infections, with cerebrovascular accidents (prevalently stroke and sinus thrombosis) occurring in 1%, myocardial infarction in 0.5% and acute limb ischemia in 0.4% of patients (Malas et al., 2020).

Aside from cerebrovascular events, other neurological complications may occur. Critically ill patients admitted to the ICU are at high risk of developing COVID-19 associated encephalopathy with delirium or confusional state (Rass et al., 2021) and, more rarely, encephalitis (Garg et al., 2021). Several sporadic cases of COVID-19-associated Guillain-Barre syndrome have been reported, with patients presenting with ascending limb weakness and potentially developing respiratory paresis requiring mechanical ventilation (Toscano et al., 2020). Following long hospital stays or ICU admissions, critical disease neuropathy or myopathy have been documented (Sharifian-Dorche et al., 2020).

After the acute infection has resolved, affected individuals may have persistent complaints or develop post-infectious complications. While not always clinically apparent, hyposmia or anosmia may persist in up to 45% of cases at three-month follow-up (Rass et al., 2021). Of patients admitted to ICU, 26% may have persistent complaints due to critical illness neuropathy/myopathy. Three months after the infection more than one-fourth of affected patients report chronic fatigue frequently associated with sleep disorders (Rass et al., 2021). Increased percentage of psychiatric disorders such as anxiety, post-traumatic stress disorder, depression or difficulty concentrating with diminished cognitive ability may also occur (Rass et al., 2021). Furthermore, follow-up of hospitalized patients at 100days has detected dyspnea or other persistent symptoms of COVID-19 in upwards of 40% of affected individuals. About 21% of patients showed reduced pulmonary diffusing capacity and 63% had persistent pulmonary alterations in computed tomography (Sonnweber et al., 2020). The frequency and duration of these symptoms are associated with the severity of the initial infection.

### Mortality

When discussing mortality rates, a distinction must be made between deaths recorded in documented, symptomatic infections (defined as case fatality rates or CFR), and estimated mortality in all infections, including asymptomatic or pauci-symptomatic infections (defined as infection fatality rates or IFR) as well as people who had a positive SARS-CoV-2 test result, while being screened after presenting due to other diseases.

Various CFR estimates for infections due to SARS-CoV-2 have been reported. These differ greatly among each other, potentially, due to the different demographics of included subjects or the respective populations or differences in healthcare resources or differences in testing strategies and capacities as well as in country-specific differences in data management and reporting. In the first published case series of the patients from the Huanan fish market outbreak, six out of 41 (15%) hospitalized subjects died; of these six, five had been previously admitted to the ICU (Huang et al., 2020). A second case series from China detected a substantially lower CFR of 4.3%, all of which had previously developed severe disease and been admitted to the ICU (Wang et al., 2020a). A further large-scale Chinese epidemiological study with 72,314 patients reported a CFR of 2.3% (Wu and McGoogan, 2020). After the infection spread to Italy, a CFR of 7.2% was calculated on the base of 22,512 cases with 1,625 deaths (Onder et al., 2020).

Infection fatality rate estimates, however, paint a somewhat more coherent picture, placing the mortality for all infections, including asymptomatic or mild disease, under 1% (Meyerowitz-Katz and Merone, 2020).

For patients with severe disease requiring ICU admission and long hospital stays, mortality is unsurprisingly significantly higher with CFRs usually in the double digits. A 2021 meta-analysis including 69 studies estimated an overall reported CFR of 45% at ICUs (Lim et al., 2021). While some studies report percentages as high as 61.5% (Yang et al., 2020), others documented a 21.7% ICU CFR (Klein et al., 2020) which may also relate to differences in criteria for ICU admission specifically with restrictions toward elderly and multi-morbid subjects (Karagiannidis et al., 2020; Wu et al., 2020). It is well known that age is a major risk factor for an increased mortality and a higher CFR; therefore, the full immunization specifically of elderly people with either mRNA or vector-based vaccines has been proven to drastically decrease CFR and the likelihood of severe cases needing ICU care by more than 90% (Haas et al., 2021).

## Diagnostics

At the time of writing, the pandemic is still ongoing and thus any patient presenting with classic flu-like symptoms or other symptoms specific for COVID-19 such as sudden occurrence of anosmia should be promptly tested for SARS-CoV-2. As the infection can be transmitted even a few days before onset of symptoms, different testing and screening strategies have been implemented in various countries to protect patients at risk, to identify potentially infected people before entry into vulnerable institutions such as hospitals or nursing homes or to enable people to take part in social activities. The efficacy of such strategies in controlling the pandemic still awaits scientific evaluation of their cost–benefit rate. Nonetheless, in order to perform an efficient contact tracing and containment of infected people and their contacts, quick, cheap, easy-to-use and accurate tests are a major need.

### Clinical Criteria

Certain telltale symptoms which raise suspicion for a SARS-CoV-2 infection include a sudden dry cough accompanied by chills, fatigue, and myalgia. While this may sound unspecific, many patients also complain of loss or alteration of taste and/or smell, as well as splitting headaches or conjunctivitis. Oftentimes, especially in the first days of the disease, the infected persons may experience gastrointestinal complaints, such as nausea, vomiting, abdominal cramps, or diarrhea. Some patients may come to medical attention due to complications of the infection, such as thrombosis or acute coronary syndrome. After the acute phase of the disease is over, patients may notice shortness of breath with exertion or at rest and, if hypoxic, panic progressing to grogginess, confusion and eventually loss of consciousness and coma (Berlin et al., 2020; Oberfeld et al., 2020). The most important sign to look out for in the early phase is fever. Later in the disease dyspnea, tachypnea, crackling rales in the pulmonary auscultation, tachycardia, and signs of cyanosis may be detected.

### Laboratory Parameters and Imaging

Analysis of blood parameters in the early phase may show signs of infection, with elevation of C-reactive protein, IL-6, or neopterin values; their values are positively correlated with disease severity and higher likelihood for ICU admission or death (Manson et al., 2020; Bellmann-Weiler et al., 2021). Importantly, low lymphocyte counts and specifically a low percentage of T-helper lymphocytes are associated with a severe course and a poor prognosis of the infection (Zhang et al., 2020b). In addition, high levels of inflammation inducible protein ferritin as well as low concentrations of the iron transfer protein transferrin and the presence of anemia upon hospital admission are linked to severe disease and poor prognosis (Bellmann-Weiler et al., 2020). Analysis of oxygen saturation of the blood by non-invasive procedures or invasive blood gas analysis is invaluable for monitoring the need for rapid therapy escalation in patients with lung disease. In patients with pulmonary involvement, thoracic CT is the diagnostic gold standard, but it must always be interpreted in context with the clinical course and result of diagnostic tests. CT can rapidly and precisely determine the extension and location of pulmonary involvement, but also provide information on superinfection with bacterial or fungal pathogens and prove/exclude pulmonary embolism. Ground-glass lesions, often bilaterally and sometimes compromising the entire lungs, are typical for SARS-CoV-2 infections. If CT is unavailable, a chest X-ray or thoracic ultrasound can also be used to estimate lung involvement (Allinovi et al., 2020).

### Confirmation of Infection

The current diagnostic gold standard is nucleic acid amplification tests (NAAT), specifically RT-PCR. This method functions by reverse-transcribing the viral RNA into complementary DNA and then amplifying several genes specific to SARS-CoV-2 until identification has occurred. The number of cycles necessary to identify the key genetic sequences is defined as the cycle threshold value (Ct-value). Therefore, samples containing more viral material will test positive with lower Ct-values than samples with very sparse viral content. While one may be tempted to correlate Ct-values with infectivity, caution exists in doing so. This is due to differences in the quality of sample acquisition, variations in laboratory procedures due to multiple site-specific factors, inter- and intra-assay variances but also based on differences in RT-PCR kits and machines produced by competing healthcare companies, which may result in diverging, not directly comparable Ct-values. Further comparison and standardization of RT-PCR for SARS-CoV-2 must occur before reliable correlation between Ct-values and infectivity may be established (Wölfel et al., 2020).

Awaiting RT-PCR test results could last for several days at the beginning of the pandemic, due to the specific test procedures but also due to logistic problems in sample acquisition and transfer to diagnostic laboratories. This negatively impacted on isolation and contact tracing strategies to control the infection, which is why improved IT concepts but also bed-site rapid RT-PCR tests resulted in a significant improvement of turnaround times. Attractive as this testing option may sound, its large-scale usage is still limited by a small number of samples which can be analyzed at the same time and by a much higher price, when compared to a “normal” but slower RT-PCR.

### Antigen Tests

During 2020, several healthcare companies developed antigen tests meant for rapid and cheaper diagnosis of subjects infected with SARS-CoV-2. These tests detect proteins produced by SARS-CoV-2 in the sampled body fluids, mainly originating from a pharyngeal or nasal swab. The sample is combined with a buffer solution and then applied to the test where viral antigens, if present, combine with specific antibodies and elicit a color reaction. Antigen tests are relatively cheap, easy to use and to interpret, and provide a result within several minutes. Nonetheless, their sensitivity is less than that of PCR tests and is dependent on viral loads, meaning that subjects with lower Ct-values in the PCR are more likely to produce a positive result in the antigen tests, whereas subjects with lower viral concentrations (high Ct-values) may not be identified by an antigen test (Lanser et al., 2021a; Liotti et al., 2021). Of note, antigen tests from different distributors may further vary in their sensitivity (Thommes et al., 2021), which is also affected by physical factors such as low or high temperature (Haage et al., 2021). Thus, antigen tests have their benefit in detecting SARS-CoV-2 infection in symptomatic persons and those with high viral loads, but their utility in the screening of asymptomatic subjects is limited by their reduced sensitivity in people with lower viral concentrations, specifically during the early onset of infection.

### Serology

Due to the relatively short incubation period after infection, detection of SARS-CoV-2 antibodies does not currently play a role in diagnosis of acute disease. In patients capable of producing anti-SARS-CoV-2 antibodies, IgM can be detected on average 5days after symptom onset and IgG already 14days after infection (Guo et al., 2020), whereas PCR tests may detect RNA due to viral shedding 2–3days before the onset of symptoms (La Marca et al., 2020; Vogl et al., 2021). However, serology testing is useful to identify people with previous infection but no or few symptoms, to estimate the prevalence of immune protection in a society for epidemiological purposes, but also to evaluate the efficacy and quality of the immune response following vaccination (Irsara et al., 2021; Klausberger et al., 2021; Vogl et al., 2021).

## Therapy

As discussed in the clinical section, most patients requiring hospital admission present with dyspnea, tachypnea, and hypoxemia. Therefore, the first and most important mainstay of treatment is supplemental oxygen if the basic peripheral oxygen saturation in the blood is below certain threshold levels, depending on the underlying diseases of the patient (Whittle et al., 2020). By supplying the lungs with additional oxygen, patients can be rendered eupneic in certain cases, greatly reducing the potential for respiratory exhaustion. For less severe cases of hypoxemia, oxygen therapy is delivered through nasal prongs. However, the amount of supplemental oxygen actually reaching the lower airways plateaus at a fraction of inspired oxygen (FiO2) of about 44% when flows in excess of 6L are used (Dondorp et al., 2020). Additional oxygen can be supplemented by nonrebreather masks, allowing for FiO2 of up to 100% when used with a 10–15L/min flow rate (Fuentes and Chowdhury, 2021). Oxygen delivery may be further increased by high flow nasal cannula (HFNC) devices, which maintain FiO2 at 100% while increasing flow to as much as 60L/min (Lodeserto et al., 2018). If patients remain hypoxemic despite maximal therapy with HFNC, they should be transferred to an IMCU or ICU for non-invasive or NIV. For stable patients, initial respiratory support is provided by non-invasive ventilation with additional positive end-expiratory pressure (PEEP) and pressure support during each inspiration, administered through airtight masks or helmets. While the additional pressure increases the oxygen flow to the lungs, PEEP increases the alveolar surface available for gas exchange and prevents atelectrauma, damage caused to the lower airways by repeated collapses and expansions (Beitler et al., 2016). Prone positioning may also increase oxygenation by reducing the abdominal viscera-mediated compression on the alveoli-rich dorsal portions of the lungs as well as atelectrauma (Coppo et al., 2020). Early application of HFNC, positive pressure ventilation, and prone positioning may decrease the need for intubation in some cases (Ding et al., 2020). However, in the event of further respiratory deterioration or exhaustion, invasive lung-protective ventilation with low tidal volume remains a life-saving option, with the alternative being prone ventilation (Botta et al., 2021).

If hypoxemia progresses despite maximal supplemental oxygen, patients may be treated with extracorporeal membrane oxygenation (ECMO). ECMO works by extracting blood from the body, oxygenating it through an artificial lung and then reinfusing it. This may be achieved by removing blood from a large-caliber vein and reinfusing it into either the venous or the arterial system; while the former is beneficial for patients with a compromised respiratory system, the latter is capable of alleviating both lungs and heart in the event of cardiopulmonary insufficiency. While the reported 40% mortality of patients needing ECMO therapy is very high (Barbaro et al., 2020), centers experienced with ECMO-technology have a chance to save the life of patients who have no other therapeutic options and who have been unresponsive to supplemental oxygen therapy and maximum invasive ventilation (Li et al., 2020).

**Figure 3:**
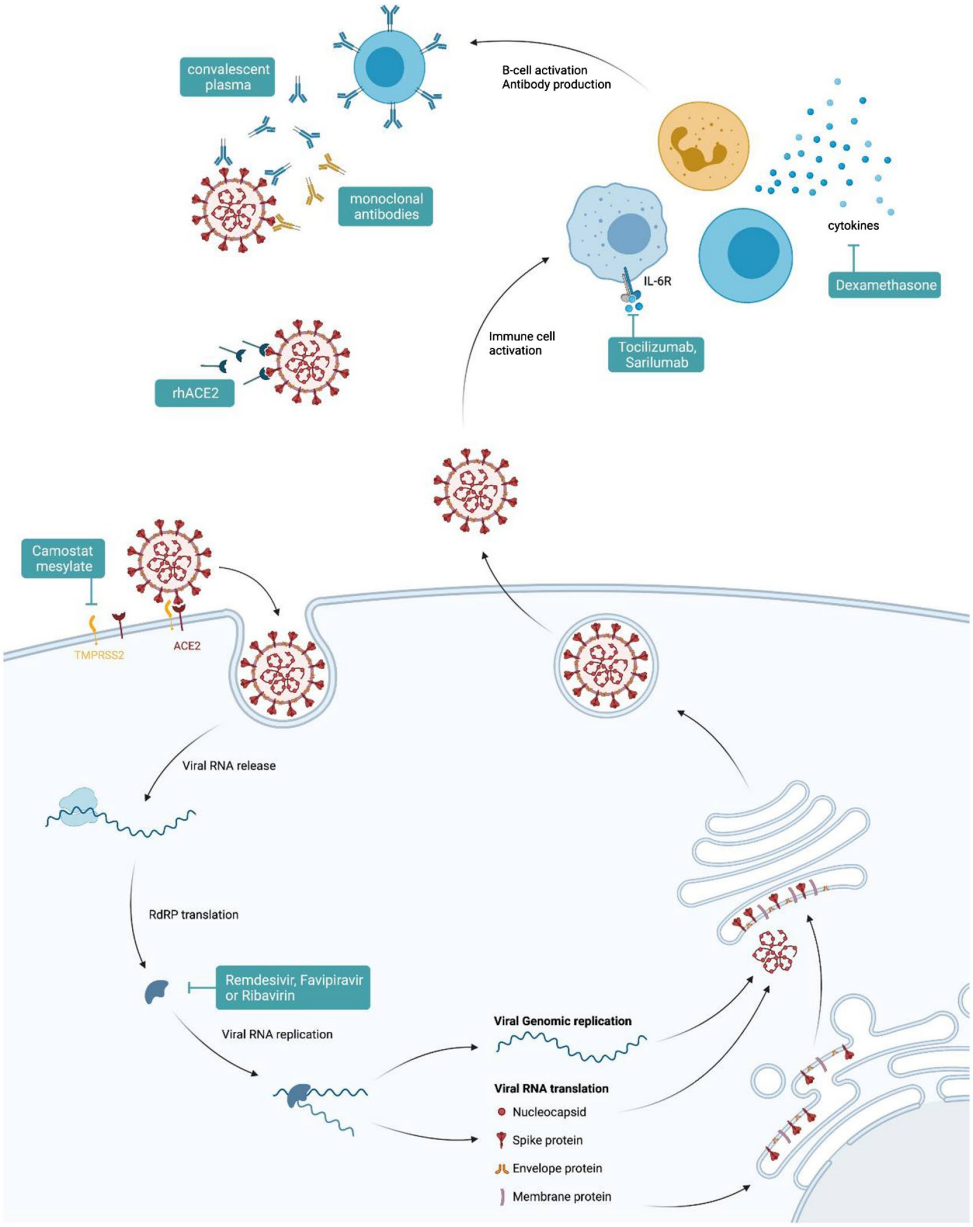
Therapeutic options for COVID-19. Graphical representation of points of attack for available therapies against severe acute respiratory syndrome coronavirus-2 (SARS-CoV-2).

Various pharmacological options for the treatment of COVID-19 have been proposed since the beginning of the pandemic (Figure 3). While no new SARS-CoV-2-specific drugs have been introduced into clinical practice thus far, various drugs have been repurposed to be studied for their effects against SARS-CoV-2 infections. We have learned that several drugs which had provided promising results from *in vitro* analyses failed to show any clinical benefit when studied in clinical trials or when analyzed in multinational consortia (Pan et al., 2021). Based on our knowledge of the pathophysiology of the disease, one very relevant point when using different drugs is their timing (Figure 1). While anti-viral medications may primarily show benefit when used early in the infection, immune-modulatory strategies should be used only in the later course of the disease when an overwhelming immune response is causing pathologic processes (Cantini et al., 2020a). Among antiviral therapies, treatment with remdesivir, an inhibitor of the RNA-dependent RNA polymerase developed in 2009 to target hepatitis C virus, Ebola virus, and respiratory syncytial virus (RSV), while not significantly influencing mortality or need for intubation, shortens the time to improvement from COVID-19 by a median of 5days as compared to no treatment (Beigel et al., 2020). Favipiravir, an orally administrable RNA-dependent RNA polymerase inhibitor originally developed to treat influenza, also shortens the time to improvement and may reduce viral shedding time (Agrawal et al., 2020). A third anti-viral drug molnupiravir, a nucleoside analogue which inhibits SARS-CoV-2 replication, has shown promising results in animal models (Rosenke et al., 2021). Both latter drugs are currently undergoing clinical efficacy evaluation in phase II and III trials. Some of the first drugs to be used against COVID-19 were the classic antimalarial drugs chloroquine and hydroxychloroquine. These substances showed *in vitro* activity against SARS-CoV-2, but failed to reduce mortality in hospitalized patients (Aapro et al., 2012; Horby et al., 2020; Wang et al., 2020c). Other drugs targeting specific viral replication mechanisms are a therapeutic mainstay of many viral diseases such as human immunodeficiency virus (HIV), influenza, or Ebola virus disease (EVD). Some of these have been repurposed to treat COVID-19. The protease-inhibiting combination of lopinavir and ritonavir originally intended for the treatment of HIV failed to improve outcomes for hospitalized patients (Cao et al., 2020).

However, many anti-viral drugs including interferons were used only on hospitalized patients, where such medication may be of limited benefit as hyperinflammation and not viral replication could be the major cause of disease at that time. In addition, viral entry inhibitors, such as camostat mesylate, TMPRSS2 blockers, or rh-ACE2, are currently being evaluated in clinical trials. The anti-parasitic drug, ivermectin, is heavily debated regarding its potential benefit for treatment of patients based on *in vitro* results and several case series which have been recently summarized (Hariyanto et al., 2021). One randomized phase II study, however, could not confirm such a benefit (Galan et al., 2021). Further randomized trials need to clarify a potential direct effect of those drugs also as a function of therapeutic timing vs. a potential indirect effect of ivermectin *via* elimination of Strongyloides in endemic areas with subsequent prevention of Strongyloides-mediated hyperinflammation. In hospitalized patients needing supplemental oxygen or mechanical ventilation, corticosteroids, such as dexamethasone, have shown benefit as they reduced mortality (Horby et al., 2021). The reduction in mortality is believed to be due to mitigation of immune system-mediated organ damage (Horby et al., 2021). Of note, mortality could not be reduced in patients without the need of supplemental oxygen at randomization (Horby et al., 2021). In an open label study, the inhalative steroid budesonide was found to reduce the frequency of hospital consultations of patients treated for SARS-CoV-2 at home; however, the true benefit of such an intervention needs to be confirmed in a high-quality randomized double blinded trial (Ramakrishnan et al., 2021). In addition, several immune modulatory drugs have been investigated for their potential to improve the outcome (Rizk et al., 2020). As an example, tocilizumab, a monoclonal antibody that blocks the receptor for the pro-inflammatory cytokine IL-6, was investigated in several trials with partly contrasting results. While some investigators found a reduction in the progression to severe disease or death, others failed to find such a benefit (Murthy and Lee, 2021; Salama et al., 2021). This indicates the need for an individualized therapeutic approach for COVID-19 patients and for finding specific algorithms or predictors for choosing between therapies. Another drug under investigation is baricitinib, a janus kinase inhibitor, used for treatment of rheumatic diseases which showed promise in small studies (Cantini et al., 2020b) but also in combination with remdesivir (Kalil et al., 2020).

Recently, numerous neutralizing monoclonal antibodies for the treatment of COVID-19 have been developed (Corti et al., 2021; Taylor et al., 2021). These showed no benefit in the treatment of hospitalized patients, but, when used early in the infection or even as a pre- or post-contact prophylaxis, they may reduce the likelihood of hospitalization or need for respiratory support in high-risk patients (Corti et al., 2021; Cruz-Teran et al., 2021), though some of the substances may exhibit limited efficacy for the emerging delta VOC 1.617 (Chen et al., 2021a). The same also holds true for convalescent plasma. Treating physicians may transfuse convalescent plasma, in the hope of inducing a passive humoral defense against the virus. However, randomized trials showed no benefit in reduction of mortality or duration of hospital stay in hospitalized patients (Janiaud et al., 2021). However, when applied within 3days after onset of symptoms, convalescent plasma therapy significantly reduced the risk for hospital admission of SARS-CoV-2-infected subjects (Libster et al., 2021). The prevention of thromboembolic complications is especially important in hospitalized patients with SARS-CoV-2 infection due to the significant pro-thrombotic inflammatory effect of the virus (Malas et al., 2020). All patients needing hospitalization and without contraindications may receive prophylactic doses of low-molecular-weight or unfractionated heparin during their hospital stay. Patients with a documented thromboembolic event should receive therapeutic dosing of anticoagulants (Bikdeli et al., 2020; Cuker et al., 2021).

More than 6,000 clinical trials are underway to determine the best possible pharmacological treatment of COVID-19. For further details on the major trials, the reader is referred to the database of publicly funded clinical studies conducted around the world (United States National Library of Medicine, 2021).

The implementation of physiotherapy and pulmonary rehabilitation during and after the disease may assist in reducing invasive ventilation and quickening the return to normal daily activities (Yang and Yang, 2020; Puchner et al., 2021).

## Prevention

### Non-pharmacological Interventions

As SARS-CoV-2 started spreading at the beginning of 2020, the WHO and the affected countries recommended or implemented various strategies to contain the further dissemination of this viral infection. In China, where the infection was first detected, dedicated hospitals were rapidly erected to isolate symptomatic COVID-19 cases from the rest of the hospitalized population. China rapidly shut down transportation in and out of affected cities and mandated lockdown measures, allowing citizens to leave their abodes only to cover basic necessities or provide assistance to those in need. This drastic lockdown affected more than 700 million people, but managed to rapidly contain the spread of the virus, dropping the number of daily new infections from thousands in January and February to under 50 in March.

Mimicking Chinese lockdown measures played a central role in many nations’ response to rising infection numbers (Baker et al., 2020). During the first large wave of the pandemic, citizens were encouraged to only leave their homes to cover their basic necessities or to assist people depending on them for survival. While those involved in the sustenance of society’s infrastructure such as healthcare personnel, service providers, and police forces were allowed to continue working, other businesses were encouraged to shut down and, where possible, implement “home office” strategies. Social contact with others was restricted to the bare minimum, such as people living together in one household or immediate coworkers.

Countries strongly affected by the pandemic temporarily closed their borders to avoid further exporting the disease (Baker et al., 2020). Certain nations enforced intra-national mobility limitations, where citizens were no longer allowed to transit between regions. So-called lockdowns, with closures of many institutions such as schools, universities, shops, restaurants, and hotels, as well as limitation of public traffic were implemented in several countries, whereas other countries followed a less stringent strategy to mitigate the infection. The efficacy of all these measures to reduce SARS-CoV-2 transmission in the population needs to be evaluated urgently in order to provide a scientific basis for future recommendations to politicians and to best combat further epidemics and pandemics.

At the interpersonal level, an almost ubiquitary mask policy has been enforced by some countries. While the decision to wear face masks was at first purely empirical and based on previous viral epidemics, mask usage managed to significantly reduce SARS-CoV-2 transmission (Mitze et al., 2020). The donning of face masks indoors became currently mandatory in almost all affected regions, and some authorities had extended the obligation even to citizens in the outdoors (Wang et al., 2020b). For general daily usage, cloth masks have been recommended to somewhat reduce the risk of transmission by both limiting the exhalation and inspiration of larger droplets, therefore reducing potential infection as well as asymptomatic spreading of viral particles. However, Asadi et al. (2020) reported no significant decrease, and on occasion even an increase, in exhaled particles while wearing a cloth mask. Surgical masks have been shown to be more effective than simple cloth masks in filtering the air we breathe, but may not be readily available to everyone (Asadi et al., 2020). For medical personnel and high-risk patients, the usage of high resistance filtered masks (N95 AKA FFP2) may significantly reduce the risk of infection (Lepelletier et al., 2020). However, it is always difficult to access the efficacy of a specific intervention such as mask wearing as many different measures were implemented at the same time. One important measure is physical distancing due to the fact that SARS-CoV-2 is mainly transmitted *via* droplets. A physical distance of at least 1m has been suggested to significantly reduce the risk of infection (Chu et al., 2020).

To reduce transmission through fomites and hand contact, hand hygiene measures, especially in the form of regular hand washing, and/or alcoholic hand rubs, have been thoroughly implemented, with dispensers often located at key points such as entrances to shops or in lavatories. Commercially available alcohol-based hand sanitizers have proven effective in inactivating SARS-CoV-2 and may therefore assist in somewhat reducing further contagion (Leslie et al., 2021). As a positive side effect, improved hand washing and mask wearing has also reduced the incidence of other respiratory viral diseases such as RSV or influenza infections.

### Vaccination

The emergence of the SARS-CoV-2 pandemic boosted the development and innovation of new vaccines. Within an incredibly short period of time, numerous vaccines with partly extraordinarily high efficacy were made available (Castells and Phillips, 2020). The first vaccines licensed by the EMA and FDA were two mRNA vaccines containing the information of the SARS-CoV-2 spike protein packed into nanoparticles, as the spike protein has been identified as the immunogenic target of the virus based on previous studies with SARS-CoV-1 (Almehdi et al., 2021). Both mRNA vaccines showed an appropriate serologic response with neutralizing antibody production after a total of two shots. The reported effectiveness in preventing infection by SARS-CoV-2 was above 95% in the respective studies (Connors et al., 2021). The side effects reported by clinical trials were relatively mild, including pain at the site of injection, gastrointestinal complaints, flu-like symptoms, headache, and fatigue. Systemic vaccination-associated reactions were more often described in younger patients and after the second vaccine dose (Polack et al., 2020; Baden et al., 2021). Of note, a large population-based survey including more than 4million vaccines in Israel demonstrated an outstanding efficacy of above 95% for prevention of infection and, among those becoming infected, a protection from severe disease or hospitalization of more than 90% (Haas et al., 2021). Meanwhile, vector-based vaccines, inactivated vaccines, and live attenuated virus vaccines have been developed and are used worldwide with varying efficacies. For further details on vaccines, indications, immunological responses, efficacy against VOCs, the reader is referred to specific recommendations of national vaccination committees or specific reviews on SARS-CoV-2 vaccination, as this topic is under ongoing investigation. One interesting aspect on vaccination originates from the observation that people who were previously infected and then received a vaccine developed higher antibody titers and improved immunological protection as compared to subjects receiving vaccines only (Krammer et al., 2021; Prendecki et al., 2021; Reynolds et al., 2021).

One important point of concern in regard to vaccination is the rare development of thrombotic thrombocytopenia, which is linked to induction of anti-platelet antibodies (PF4), whereas inflammation induced by the infection or the vaccine itself also increases the risk for thrombotic events (Fontana et al., 2020; Mei et al., 2020; Greinacher et al., 2021; Schultz et al., 2021).

## Conclusion

In this review, we attempt to provide the readers with an overview on the complex and ever-mutating topic of COVID-19. A limitation of this work is the dynamic nature of the still evolving pandemic, with new variants of concern being discovered almost monthly and multiple medications and vaccines being tested and produced at hitherto unfathomable speeds. Due to the enormous amount of available literature, at times with contradicting recommendations, it is imperative that future researchers strive to clarify the results by unifying the data with meta-analysis and other systematic reviews.

## Author Contributions

FB and GW conceptualized the project, drafted the manuscript, and performed literature search. LL performed the graphics (created with BioRender). LL and RB-W gave intellectual input and critically edited the manuscript. GW was responsible for supervision. All authors contributed to the article and approved the submitted version.

## Conflict of Interest

The authors declare that the research was conducted in the absence of any commercial or financial relationships that could be construed as a potential conflict of interest.

## Publisher’s Note

All claims expressed in this article are solely those of the authors and do not necessarily represent those of their affiliated organizations, or those of the publisher, the editors and the reviewers. Any product that may be evaluated in this article, or claim that may be made by its manufacturer, is not guaranteed or endorsed by the publisher.
